# The Ages and Stages Questionnaire: Social-Emotional—What Is the Optimal Cut-Off for 3-Year-Olds in the Swedish Setting?

**DOI:** 10.3389/fped.2022.756239

**Published:** 2022-02-09

**Authors:** Masoud Vaezghasemi, Eva Eurenius, Anneli Ivarsson, Linda Richter Sundberg, Sven Arne Silfverdal, Marie Lindkvist

**Affiliations:** ^1^Department of Epidemiology and Global Health, Umeå University, Umeå, Sweden; ^2^Department of Clinical Science, Pediatrics, Umeå University, Umeå, Sweden

**Keywords:** emotional and behavioral problems, mental health, preschool children, screening, Strengths and Difficulties Questionnaire (SDQ), Receiver Operating Characteristic (ROC) analysis

## Abstract

**Objective:**

Expressions of emotional and behavioral symptoms in preschool age can predict mental health problems in adolescence and adulthood. The Ages and Stages Questionnaires: Social-Emotional (ASQ:SE) has been successful in detecting social and emotional problems in young children in some countries but had not been tested in Sweden. The objective of this study was to determine the optimal cut-off for the ASQ:SE instrument when administered to 3-year-old children in a northern Swedish setting, using the Strengths and Difficulties Questionnaire (SDQ) as the reference.

**Methods:**

The ASQ:SE (36-month interval, first edition) was administered at routine 3-year-olds' visits to Child Health Care centers in Region Västerbotten, Sweden. During the study period (September 2017 to March 2018) parents were invited to also fill out the SDQ (2–4 year version). In the final analyses 191 children fulfilled the criteria for inclusion in the study sample. Non-parametric Receiver Operating Characteristic analysis was performed to quantify the discriminatory accuracy of ASQ:SE based on SDQ.

**Results:**

The Pearson correlation between ASQ:SE and SDQ indicated strong correlation between the two instruments. The Receiver Operating Characteristic curve showed good accuracy of ASQ:SE in relation to SDQ. However, our results suggest that the existing ASQ:SE cut-off score of 59 was not optimal in the Swedish context. Changing the cut-off from 59 to 50 would allow us to detect 100% (*n* = 14) of children with problems according to SDQ, compared to 64% (*n* = 9) when the cut-off was 59. However, the proportion of false positives would be higher (9% compared to 3%).

**Conclusion:**

The main finding was that for 3-year-olds in Sweden a decreased ASQ:SE cut-off score of 50 would be optimal. This would increase the detection rate of at-risk children according to SDQ (true positive), thus prioritizing sensitivity. Our conclusion is that, although this change would result in more false positives, this would be justifiable.

## Introduction

Expressions of emotional and behavioral symptoms in preschool age children can predict mental health problems in adolescence (1) and adulthood (2). Research shows that supporting early social-emotional development can lead to positive outcomes in mental health, education, and employment, and a lower likelihood of criminal activity and substance abuse in later life (3). These findings highlight the need for methods which can detect vulnerabilities in children's social and emotional functioning.

Developmental screening tools are designed to identify children with potentially delayed or atypical development. However, there is no universally accepted screening tool appropriate for all populations and all ages. Population characteristics and health care providers' preferences determine the choice and suitability of the instrument (4). A screening tool requires well-established psychometric properties, including validity and reliability, so that researchers, providers, and care takers can have confidence in what is being measured. Moreover, the instrument's accuracy in identifying children at risk (sensitivity) or not at risk (specificity) is important for the context in which the instrument is being used (5). A cut-off resulting in false negatives can deprive children from receiving appropriate preventive or curative measures. Alternatively, a cut-off resulting in false positives can waste resources and lead to unnecessary stigmatization (6).

The Ages and Stages Questionnaires: Social-Emotional (ASQ:SE) which was developed to screen social-emotional competencies and problems, has shown adequate psychometric properties (7–9). Many studies support the instrument's easy administration, short completion time, simple interpretation, and capacity to enhance the clinician's ability to detect children at risk of developmental delays in social and emotional skills (10–14). Despite the broad and popular use of the ASQ:SE internationally, we are not aware of any attempts to evaluate whether the cut-off score, based on the United States (US) population, is optimal for detecting social-emotional problems in children in Sweden.

The ASQ:SE was introduced in routine Child Health Care (CHC) services in Region Västerbotten in Sweden in 2014 but uses a cut off score derived from settings outside Sweden. The Strengths and Difficulties Questionnaire (SDQ) is a common tool for identifying mental health problems in children and adolescents internationally and in Sweden (15–25). The aim of this study is to find an optimal cut-off for the use of the ASQ:SE instrument among 3-year-old children in Sweden, using the SDQ instrument as the reference.

## Materials and Methods

### Study Context

Through a repeated cross-sectional study design, the CHC services in Region Västerbotten, Sweden, routinely collect data on 3-year-old children by administration of the ASQ:SE. This is carried out through collaboration with the Salut Child Health Programme, which involves a universal multisectoral health promoting intervention (26). The ASQ:SE is used as part of a staff–parent dialogue aimed at increasing awareness of children's social and emotional development and identifying children who might benefit from extra support. Out of 40 eligible CHC centers in Region Västerbotten, 12 centers with 21 nurses agreed to recruit participants, and assist in data collection. Prior to the regular 3-year-old visit, an invitation letter as well as questionnaires were sent to parents' home address through postal service. Parents were asked to fill-out the questionnaires and bring them along when they visit CHC. The participating centers are geographically spread across the Region including both rural and urban areas.

### Study Participants

The data collection period extended from September 2017 to March 2018, with one CHC center continuing until June 2018. There were 300 3-year-old children who were invited. The questions in the Swedish versions of both the ASQ:SE and SDQ were answered by the parents of 246 children (82%). Of these, 191 children were included in the final analyses (64%) after excluding 55 children, of whom 54 were aged outside the required range (33–41 months) (9), and one because of missing information on sex. The questionnaires were completed by either the parents jointly (63%), by mothers alone (35%) or by fathers alone (2%).

### Measurements

#### Ages and Stages Questionnaires: Social-Emotional (ASQ:SE)

The ASQ:SE is designed for the reporting of social-emotional competencies and problems among children aged 3–63 months by parents or nominated adults. The instrument, first published in 2002, has undergone extensive psychometric tests in the US context (7–9). The second edition was published in 2015 (27). We used the Swedish translation of the 36-month version of the first edition of ASQ:SE (9) according to established recommendations (28). The age span for the 36-month version ranged from 33 months and 0 days to 41 months and 29 days in accordance with the ASQ:SE User's Guide (9). The instrument comprises 34 items of which the last three are open-ended questions and are not used in the present study. For 31 of the items, the parent indicates on a three-point Likert scale (0, 5, or 10 points) how often they perceived the stated behavior of their child (always or often, sometimes, seldom, or never) and whether this behavior was of concern for them (5 points). This gives a total score of between zero and 465, where, based on US evidence, a score of 59 or above indicates social-emotional problems (9).

#### Strengths and Difficulties Questionnaire (SDQ)

The SDQ is available for parents' and teachers' assessments of children and adolescents internationally (17) as well as in Sweden (22). The Swedish version of the SDQ has shown adequate psychometric properties (25). It has also been validated for parental use among children (24) and adolescents (23). The preschool version of SDQ has been validated in the United Kingdom as a tool for identifying 3- to 4-year-olds with emotional and behavioral difficulties (19). In Sweden, acceptable construct validity was also concluded for parents' and teachers' ratings of preschool children (21). In addition, the Swedish translation demonstrated good psychometric properties in a normative sample of preschool children with parents and teachers as the respondents (20).

SDQ consists of 5 scales with 5 items each, giving 25 items in total. The parent indicates on a three-point Likert scale (0, 1, or 2 points) the extent to which the stated behavior is relevant for their child (not true, somewhat true, certainly true). This results in a total difficulty score of 0–40 points, by summing scores from four of the scales (except the last prosocial scale), i.e., 20 of the 25 items. A score of 13 or above has been suggested as the cut-off for behavior problems based on studies from United Kingdom (UK) (29). This score was confirmed as an appropriate cut-off for children in Sweden (expressed as above 12) (20).

### Data Analysis and Statistical Considerations

#### Descriptive Analysis

We used frequencies, percentages, ranges, median, mean and standard deviations (SD) to report the distribution of both the ASQ:SE and SDQ. Statistical differences between boys and girls were examined by Independent Samples *t*-tests, Wilcoxon rank-sum test and Pearson chi-square tests. *P*-values were considered significant at the level of 0.05.

#### Relationship Between ASQ:SE and SDQ

A scatterplot was used to illustrate the relationship between ASQ:SE and SDQ. Pearson and Spearman correlation methods quantified the relationship between the two instruments.

#### Receiver Operating Characteristic (ROC) Analysis

We performed non-parametric Receiver Operating Characteristic (ROC) analysis to quantify the discriminatory accuracy of ASQ:SE based on the SDQ score at or above the Swedish cut-off score of 13. The analysis gives Bamber and Hanley confidence intervals (CI) for the area under the ROC curve, which illustrates the ability of the test to discriminate. The ROC curve is a plot of the diagnostic test's sensitivity vs. specificity. The sensitivity is the fraction of cases with the disease that are correctly classified by the diagnostic test, whereas the specificity is the fraction of cases without the disease that are correctly classified. Thus, the sensitivity is the true-positive rate, and the specificity is the true-negative rate, and the best cut-off is the score that maximizes both. The positive predictive value (PPV) and the negative predictive value (NPV) are related measures. PPV is the fraction of cases having the disease if the diagnostic test is positive and NPV is the fraction of cases not having the disease if the test is negative. Investigation of a new Swedish cut-off score for ASQ:SE was performed by analyzing sensitivity, specificity, PPV, and NPV for two different cut-off sores in the ASQ:SE using the SDQ UK cut-off. Analyses were performed using STATA/SE version 16.1 (StataCorp, College Station, TX, USA) and MedCalc for Windows, version 19.4 (MedCalc Software, Ostend, Belgium).

#### Sample Size and Power Calculation

Based on 80% power, 0.05 significance, an allocation ratio 10 (one case above the cut-off per 10 children) and the area under the ROC curve equal to 0.7, a sample of at least 143 children was needed for this study.

### Ethics

Parental consent was required for participation. Our research using ASQ:SE was approved by the Regional Ethical Review Board in Umeå (2013-268-31 Ö). The supplementary collection of data using the SDQ was approved in an amendment to this application (2017/124-32).

## Results

The mean age of the 191 children in the sample was 36 months (range = 33–41). There were 95 boys and 96 girls.

### ASQ:SE and SDQ Description

Among the 3-year-olds, 7.3% had indications of social-emotional problems based on ASQ:SE when using the US cut-off (≥59), and equally many (7.3%) had indications of behavior problems based on SDQ (≥13), although they were not exactly the same children. Further descriptive results are shown in Table 1.

**Table 1:** Descriptive of ASQ:SE^a^ and SDQ^b^ in a population-based study of 3-year-olds.

**Instrument**			**Children**
			***n* = 191**
ASQ:SE^a^ 36-month interval	Total score	Range	0–230
		Median (IQR)	20 (10–40)
		Mean (SD)	27.0 (29.5)
	Above the	US cut-off (≥59), n (%)	14^c^ (7.3)
SDQ^b^ 2–4 year version	Total score	Range	0–28
		Median (IQR)	5 (3–8)
		Mean (SD)	5.9 (4.2)
	Above the	UK cut-off (≥13), n (%)	14^c^ (7.3)

a *ASQ:SE, Ages and Stages Questionnaires: Social-Emotional, first edition*.

b *SDQ, The Strengths and Difficulties Questionnaire*.

c *Note, that these 14 children are not exactly the same children for ASQ:SE and SDQ*.

### Relationship Between ASQ:SE and SDQ

**Figure 1:**
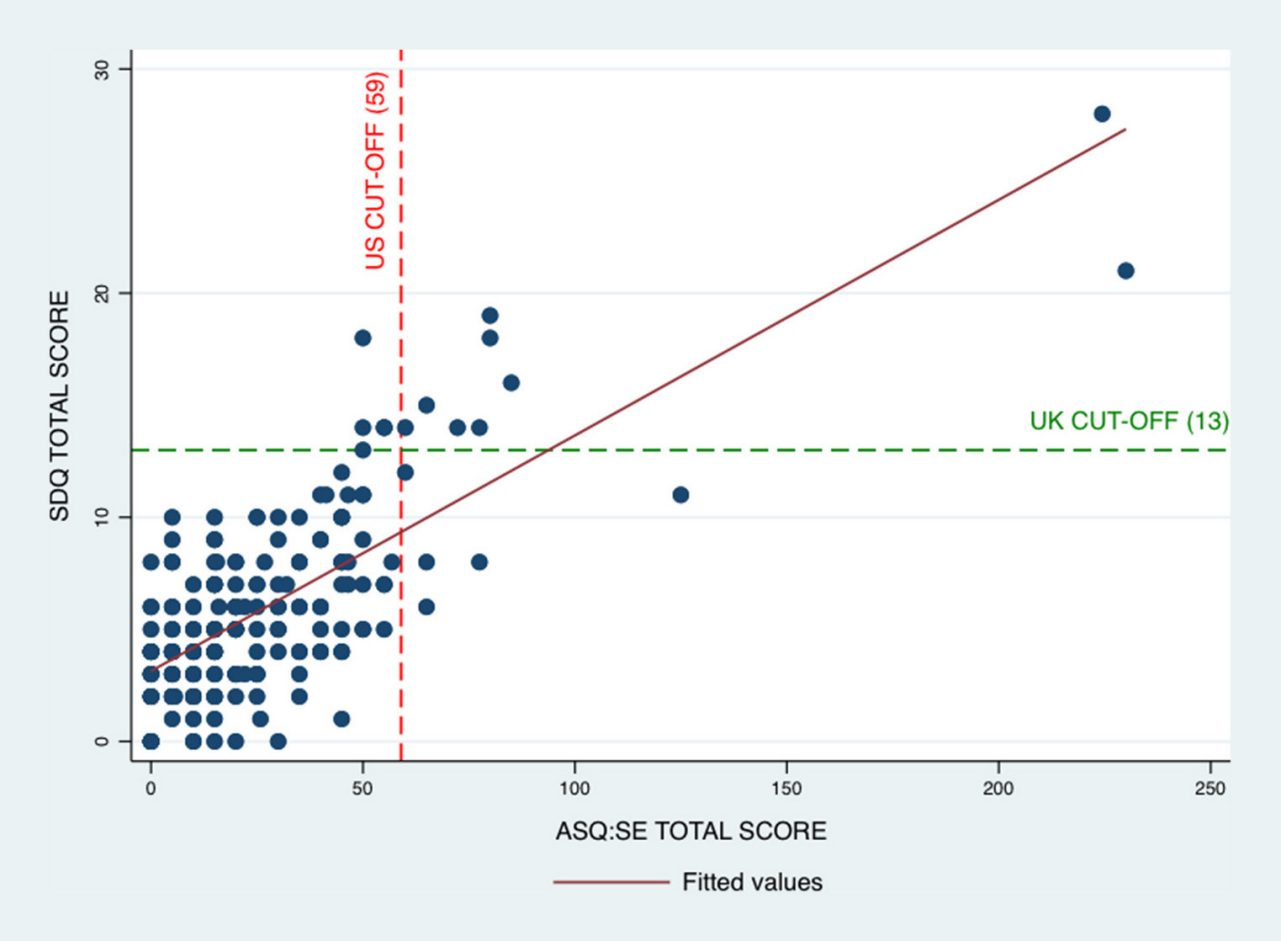
The correlation between ASQ:SE and SDQ in a population-based study of 3-year olds. ASQ:SE, Ages and Stages Questionnaires: Social-Emotional for 36-month interval, first edition; SDQ, Strengths and Difficulties Questionnaire, 2–4 year version.

A scatterplot showing the relationship between ASQ:SE and SDQ is presented in Figure 1. The Pearson correlation value of 0.73, indicated strong correlation between the two instruments. A sensitivity analysis with the Spearman correlation statistic yielded a value of 0.60, thereby showing correlation.

### Receiver Operating Characteristics (ROC) Curve and Corresponding Measures

**Figure 2:**
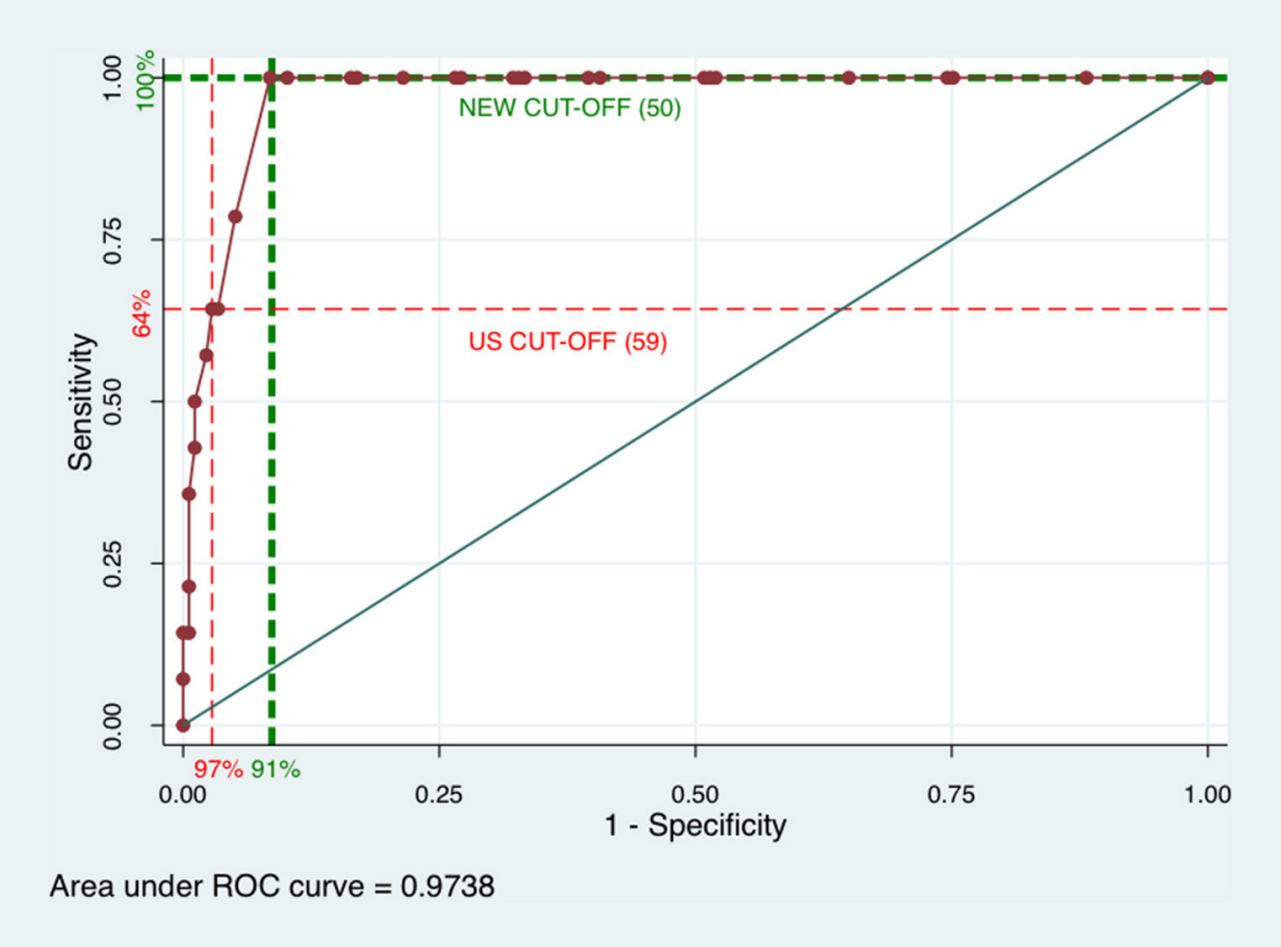
Receiver Operating Characteristics (ROC) curve for ASQ:SE and SDQ in a population-based study of 3-year olds. ASQ:SE, Ages and Stages Questionnaires: Social-Emotional for 36-month interval, first edition; SDQ, Strengths and Difficulties Questionnaire, 2–4 year version.

The calculation of the area under the ROC curve resulted in the value 0.97 (CI = 0.95–0.99), suggesting good accuracy in the ASQ:SE in relation to the SDQ (Figure 2). The highest sum of ASQ:SE sensitivity and specificity were found for the cut-off score of 50. If 50 is the new ASQ:SE cut-off in Sweden, we would expect to detect 100% (*n* = 14) of those children with problems according to SDQ, compared to only 64% (*n* = 9) with a cut-off score of 59 (Table 2). On the other hand, with this new cut-off, 9% (*n* = 15) of children without problems would be identified as having problems compared to 3% (*n* = 5) when the cut-off is 59 (i.e., false positive). Comparison of predicted values for the two cut-offs shows a similar pattern. Decreasing the ASQ:SE cut-off to 50, compared to the US cut-off score of 59, can lead to the situation in which all children with scores below the ASQ:SE cut-off, also have SDQ scores below the cut-off, i.e., children with no problems according to either the ASQ:SE or SDQ. However, the proportion with a true positive ASQ:SE score according to the SDQ would decrease from 65 to 49%.

**Table 2:** ASQ:SE^a^ performance with different cut-off scores and using SDQ^b^ as comparison in a population bases study of 3-year-olds.

	**ASQ:SE cut-off**
	**≥50**	**≥59**
Cases detected, *n* (%)	14 (100)	9 (64)
Sensitivity % (95% CI)^c^	100 (77–100)	64 (35–87)
Specificity % (95% CI)	91 (86–95)	97 (94–99)
Positive predicted value % (95% CI)	49 (37–61)	65 (42–83)
Negative predicted value % (95% CI)	100 (97–100)	97 (94–99)

a *ASQ:SE, Ages and Stages Questionnaires: Social-Emotional for 36-month interval, first edition*.

b *SDQ, Strengths and Difficulties Questionnaire 2–4 year version, using the UK cut-off ≥13*.

c *CI, Confidence Interval*.

## Discussion

### The Main Findings

The ASQ:SE is used worldwide for assessing social and emotional development in children. This is the first attempt to find an optimal cut-off in a Swedish context and in a general population of 3-year-olds. We showed that the instrument's US cut-off score of 59 was not the best choice for 3-year–olds in Sweden. Decreasing the cut-off to 50 would increase the detection rate of children at risk of problems according to the SDQ, but at the same time a larger proportion of those children would be falsely positive, and thus, incorrectly considered at risk of social-emotional problems. This lowering of the cut-off would prioritize sensitivity, which is what should be favored in a screening situation (30).

Sensitivity (the fraction of positive cases that are correctly classified) and specificity (the fraction of negative cases that are correctly classified) depend on how the cut-off score in the comparator instrument is defined. The selection of cut-off scores always involves a trade-off between sensitivity and specificity. The choice depends largely on the context and population for which the instrument is intended. In this study the UK cut-off for the instrument SDQ is used as our standard for “correctly classified children.” The SDQ cut-off was recently confirmed appropriate for Swedish children (20).

By lowering the ASQ:SE cut-off to 50 we would expect to detect more children above the SDQ cut-off. We see this an advantage because the two instruments capture somewhat different types of problematic behaviors. On the other hand, the positive predicted value for ASQ:SE cut-off score of 50 tells us that half of the children above the cut-off would not have problems according to the SDQ. This can lead to the identification of more children with a larger range of social-emotional problems (according to both SDQ and ASQ.SE). In that case it is possible that the health system in Sweden would not have the capacity to respond to all children and families identified as vulnerable. Thus, evidence-based knowledge gained through studies such as this, may serve as a basis for developing policies that support allocating more resources for the benefit of children's mental health.

### Comparison With Other Studies

A 2016 review which investigated psychometric properties of the ASQ:SE in children aged between two and two and a half years, found that reliability, sensitivity, and specificity were generally good for the original version of ASQ:SE, but the properties for translated/adapted versions were not consistent (31). The conclusion was that it is important to consider contextual factors when measuring child development using the ASQ:SE. Another review with the objective of examining the classification accuracy of measures of overall psychopathology recommended for pediatric primary care screening. The results showed that ASQ:SE can produce high levels of sensitivity and specificity using the original US cut-off score, although the number of included studies was limited (32). Both reviews found that the screening measure Child Behavior Checklist (CBCL) (33) was the primary criterion measure. However, CBCL is more focused on psychopathology which was one of the reasons for the choice of the SDQ in this study.

A 2018 Norwegian study that validated a teacher completed ASQ:SE against the Caregiver-Teacher Report Form (C-TRF) among 5-year-old children, concluded that the ASQ:SE had good screening accuracy in detecting children at risk for social and emotional problems (34). However, the authors proposed a reduction in the ASQ:SE cut-off scores for 18- and 24-month versions in the Norwegian context arguing that this would increase the detection rate of children with social-emotional problems (true positives). In contrast, they suggested a higher cut-off for the ASQ:SE 36-month interval, compared with the cut-off recommended for the US population of the same age.

### Strengths and Limitations

A major strength of the study was the continuous data collection of ASQ:SE for 3-year-old routine visits within CHC. It was also important that nurses in these clinics had administered the ASQ:SE for several years. Our sample was a sub-sample of the total number of families linked to the Salut Child Health Promotion Programme. The proportion of 3-year-olds with social-emotional problems in this sub-sample was smaller (7.3%) compared to the total population (9.0%) in Region Västerbotten (35). The lower prevalence could be because individuals (in this case parents) who agreed to answer both questionnaires in studies might be more privileged (e.g., more interested in the specific topic and have higher education or income) than other individuals (36). Socioeconomically disadvantaged children are more likely to develop mental health problems which means that the children in this study might be less vulnerable to mental health problems (37). In addition, the questionnaires were only in Swedish, therefore, non-Swedish speaking parents were not included. We believe this potential sampling bias will not deter our results, as we did not aim to investigate whether ASQ:SE or SDQ discriminate between different groups. We rather aimed to compare the ASQ:SE and SDQ for the same children regardless of their background characteristics. Another limitation could be our choice of the SDQ questionnaire for comparison with ASQ:SE. However, we decided to use SDQ, because it is reported to be a good tool for identification of psychosocial problems in preschool children (38) and it is widely used in Sweden. Although, one may argue that a more assessment-based or diagnostic-based instrument such as the CBCL might be more relevant for determining the optimal cut-off score for ASQ:SE. Assessing social, emotional, and mental health among children can be complex and involve a variety of assessments in order to gain a comprehensive understanding of the situation. Future studies may shed a better light on the issue of the cut-off score by evaluating the ASQ:SE cut-off against clinical assessments and/or observations of the child's social and emotional functioning and behaviors. In addition, in this study we have used the original ASQ:SE published in 2002. In future work on children's social-emotional health we will strive to use an up-to-date, culturally adopted, Swedish translated version of the ASQ:SE-2 (27).

### Clinical and Policy Implications

It seems feasible to use the ASQ:SE for identifying Swedish preschool children's social-emotional problems reported by parents, and to decrease the US cut-off score of 59 to 50. CHC nurses collate responses, discuss items of parental concern, and help identify children in need of further professional monitoring and extra support. As a next step, consultation between the parent(s) and a child specialist may help to target health care to those most in need. This is already done today in Region Västerbotten by either the CHC nurse or a psychologist, speech therapist, physician, or other health professional. The choice of cut-off for detecting children with social-emotional problems at 3-years of age is of utmost importance for clinical and ethical reasons. A more accurate and validated cut-off would give health care professionals and parents more confidence in using ASQ:SE in the Swedish context. In addition, this implies that the premise of the instrument ASQ:SE can adequately be realized, i.e., that the “the right” children and families are identified and potentially receiving preventive, supportive or curative interventions. It should be noted that the majority of children, also those identified with an ASQ:SE above any of the cut-offs, may not necessarily need an expensive, comprehensive follow-up evaluation and intensive services. Many can likely be helped by receiving a “baseline,” lower-cost intervention as discussed above. Revaluation of the presently suggested lowering of the ASQ:SE cut-off from 59 to 50 will be needed if this change results in overwhelming the health care and social welfare systems. However, importantly, there will be a need to re-evaluate the ASQ:SE cut-off before a Swedish version of the ASQ:SE-2 is implemented, and it should preferably be done using clinical assessments and/or observations as described above. Future research should consider investigating the determinants of children's social-emotional problems during pregnancy, birth and early childhood to help prevent mental health problems developing in later life. In addition, Swedish policymakers could advocate population-wide implementation of the ASQ:SE along with the follow-up of vulnerable children at risk. By highlighting these issues, we hope that this paper will contribute to a future in which the mental health of preschool age children is given high priority.

## Conclusion

ASQ:SE is a useful instrument for use in routine CHC at the 3-year old visit in Sweden. In the Swedish context we suggest decreasing the cut-off to 50 (compared to the US cut-off of 59) as this prioritizes sensitivity as recommended by WHO and others. Our findings illustrate the importance of deriving country specific cut-offs.

## Data Availability Statement

The datasets presented in this article are not readily available because Region Västerbotten originally collected the data for a child health survey (https://www.regionvasterbotten.se/salut). We accessed data for the present study after approval from both the Region Västerbotten and the Ethical Vetting Board. The data are not publicly available but access for replication analyses is possible. Requests to access the datasets should be directed to https://www.regionvasterbotten.se/salut.

## Ethics Statement

Parental consent was required for participation. Our research using ASQ:SE was approved by the Regional Ethical Review Board in Umeå (2013-268-31 Ö). The supplementary collection of data using the SDQ was approved in an amendment to this application (2017/124-32). Written informed consent to participate in this study was provided by the participants' legal guardian/next of kin.

## Author Contributions

MV and ML conceived and designed the study, carried out the statistical analyses, and prepared the first draft. AI, LR, and EE were responsible for selecting what instruments to use. EE, LR, and SAS organized the data collection. LR contributed with interpretation of results from the psychometric questionnaires. All authors contributed to the writing process and have approved the final manuscript.

## Funding

This study was supported by the Public Health Agency of Sweden, Region Västerbotten, and Umeå University.

## Conflict of Interest

The authors declare that the research was conducted in the absence of any commercial or financial relationships that could be construed as a potential conflict of interest.

## Publisher's Note

All claims expressed in this article are solely those of the authors and do not necessarily represent those of their affiliated organizations, or those of the publisher, the editors and the reviewers. Any product that may be evaluated in this article, or claim that may be made by its manufacturer, is not guaranteed or endorsed by the publisher.
